# MiR-93-5p enhances growth and angiogenesis capacity of HUVECs by down-regulating EPLIN

**DOI:** 10.18632/oncotarget.22300

**Published:** 2017-11-06

**Authors:** Liang Liang, Lei Zhao, Ying Zan, Qing Zhu, Juan Ren, Xinhan Zhao

**Affiliations:** ^1^ Department of Oncology, The Second Affiliated Hospital, Medical School of Xi’an Jiaotong University, Xi’an 710004, China; ^2^ Department of Molecular Physiology and Biophysics, University of Iowa, Carver College of Medicine, Iowa City, IA 52242, USA; ^3^ Department of Radiation Oncology, The First Affiliated Hospital, Medical School of Xi’an Jiaotong University, Xi’an 710061, China; ^4^ Department of Oncology, The First Affiliated Hospital, Medical School of Xi’an Jiaotong University, Xi’an 710061, China

**Keywords:** miR-93-5p, EPLIN, angiogenesis, HUVECs, TNBC

## Abstract

Tumor angiogenesis is essential in delivering oxygen and nutrients to growing tumors, and therefore considered as a hallmark of cancer. MicroRNAs (miRNAs) have been shown to play important roles in regulating tumor angiogenesis. MicroRNA-93-5p (miR-93-5p) has been identified as an oncogenic miRNA in a variety of human malignancies and involved in tumor angiogenesis in astrocytoma. However, the direct effect(s) of miR-93-5p on the biological behaviors of endothelial cells have not been investigated. Thus, in the present study we investigated the role(s) of miR-93-5p in regulating the functions of human umbilical vein endothelial cells (HUVECs). We found that triple negative breast cancer (TNBC) tissues with higher levels of miR-93-5p showed higher blood vessel density. Overexpression of miR-93-5p accelerated HUVECs proliferation and migration and promoted HUVECs lumen formation and sprouting *in vitro*, while blockade of miR-93-5p suppressed HUVECs migration and angiogenic capacity. The mechanistic studies revealed that miR-93-5p can promote angiogenic process through inhibiting epithelial protein lost in neoplasm (EPLIN) expression in HUVECs. In sum, our results have indicated that miR-93-5p promoted angiogenesis through down-regulating EPLIN and therefore represented a promising target for developing novel anti-angiogenic therapeutics.

## INTRODUCTION

Tumor angiogenesis is essential in delivering oxygen and nutrients to growing tumors, and therefore considered as a hallmark of cancer [1]. Angiogenesis has been shown to play critical roles in throughout all phases of cancer formation, aggression, invasion and metastasis [2]. Given the importance of angiogenesis in cancer development, in recent years, inhibition of tumor angiogenesis in line with conventional therapies has become an effective anti-cancer strategy against several types of cancers, such as colorectal cancer [3], kidney cancer [4] and liver cancer [5]. Unfortunately, the clinically approved anti-angiogenic reagents are only effective in specific cancer types and in a subset of patients, and many who are initially responsive develop resistance over time [6]. In addition, some primary and metastatic tumors can develop and progress in the absence of angiogenesis, adding another layer of complexity to developing anti-angiogenic therapeutics [6]. Therefore, there is an urgent need to better understand the molecular basis of cancer angiogenesis for developing novel therapeutic strategies against newly formed cancer vasculatures.

MicroRNAs (miRNAs) are a family of short non-coding RNAs that regulate gene expression by binding to the 3’ untranslated region (3’UTR) of mRNAs [7, 8]. Accumulating evidence has shown that miRNAs are involved in the process of angiogenesis via regulating the expression levels of pro- or anti-angiogenic factors [9]. MiR-93-5p, a member of the miR-106b∼25 cluster, has been significantly up-regulated in multiple types of cancers, including breast cancer [10], osteosarcoma [11] and gastric cancer [12], and functions as an onco-miRNA in tumor growth [10–12]. miR-93-5p has also been reported to participate in regulating angiogenesis in cancers. Fang *et al.* reported that miR-93-5p promoted tumor angiogenesis and metastasis by suppressing LATS2 expression in astrocytoma [13]. In another study, Fang *et al.* showed that miR-93 promoted astrocytoma growth and angiogenesis by down-regulating integrin-β8 [14]. Fabbri *et al.* reported that miR-93 regulated the secretion of a panel of cytokines, chemokines and growth factors that were involved in angiogenesis in gliomas through suppressing IL-8 and VEGF [15]. However, in these studies, miR-93-5p expression level was either up- or down-regulated in cancer cell lines, which were then co-cultured with endothelial cells (ECs) to examine the effect(s) of miR-93-5p on angiogenic capacities of ECs. No reference regarding the direct effect(s) of miR-93-5p on angiogenesis of ECs has been published.

Epithelial protein lost in neoplasm (EPLIN) is a cytoskeleton-associated protein involved in regulating actin dynamics and cell motility. Sanders *et al.* showed that overexpression of EPLIN in HECV endothelial cells resulted in a significant reduction in cell migration and tubule formation [16]. In addition, the 3’-UTR of *EPLIN* mRNA has the targeting sequence of the seed region of miR-93-5p and EPLIN has been experimentally validated as a target of miR-93-5p by several cross-linking immunoprecipitation (CLIP)-based high throughput experiments.

We have reported that miR-93-5p played an oncogenic role in triple negative breast cancer (TNBC) development [17]. Given the pro-angiogenic effect of miR-93-5p in glioma, we therefore investigated whether miR-93-5p was involved in regulating angiogenesis in TNBC and further explored the underlying mechanism(s). In this study, we have demonstrated that TNBC tissues with up-regulated miR-93-5p level also had a higher vessel density. Overexpression of miR-93-5p accelerated human umbilical vein endothelial cells (HUVECs) proliferation and migration and promoted their lumen formation and sprouting *in vitro*, while blockade of miR-93-5p led to suppression in migration and angiogenic response of HUVECs. In addition, the mechanistic studies have revealed that miR-93-5p promoted the angiogenesis process through inhibiting EPLIN expression in HUVECs. In sum, our results have indicated that miR-93-5p promoted angiogenesis through down-regulating EPLIN and represented a promising target for developing novel anti-angiogenesis therapeutics.

## RESULTS

### miR-93-5p expression level was positively correlated with vessel density in breast cancer

**Figure 1:**
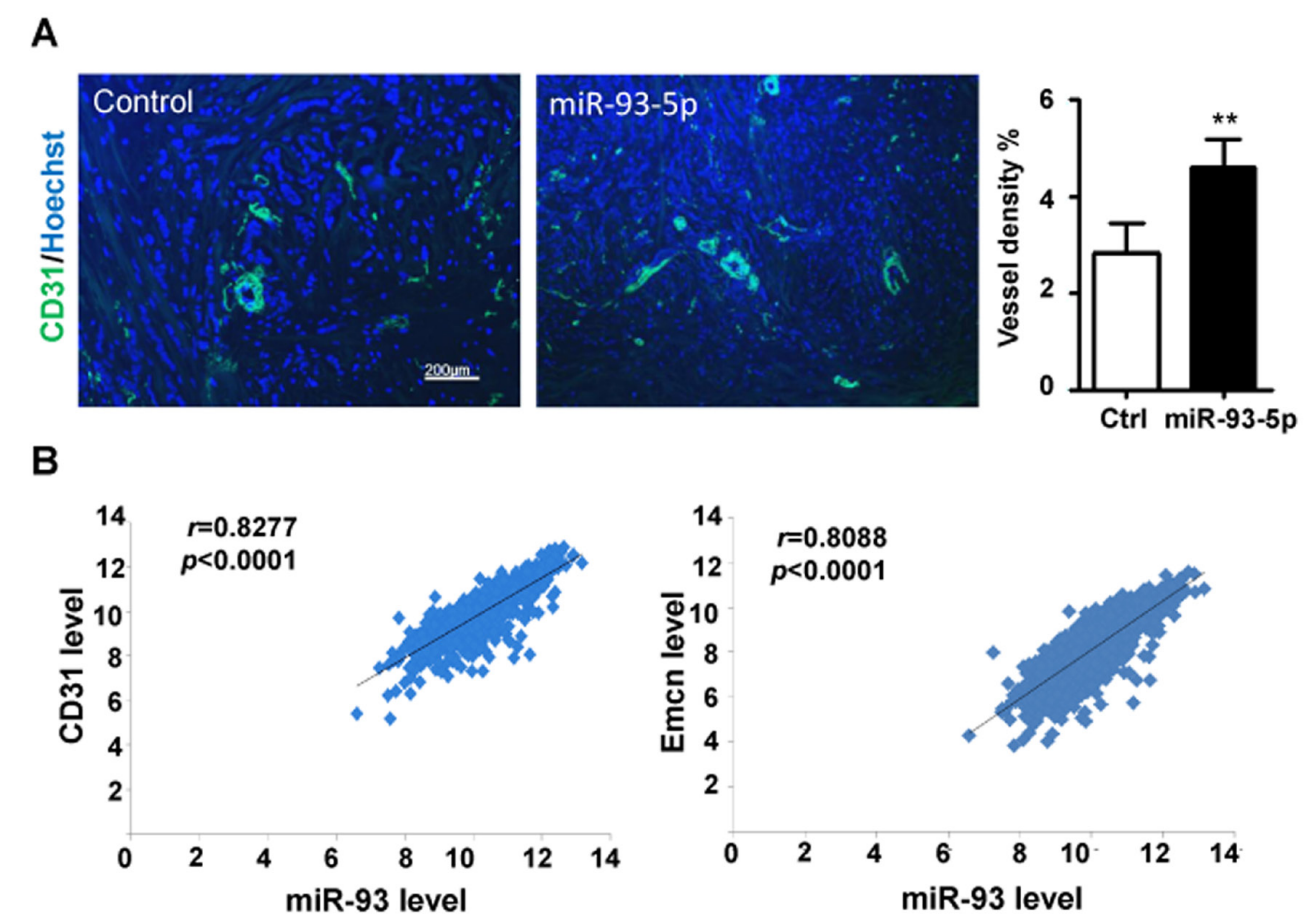
miR-93-5p expression level was positively correlated with blood vessel density in breast cancer **(A)** TNBC tissues were stained for CD31 and Hoechst, then analyzed by a fluorescence microscope, and the percentages of area with positive CD31 were calculated. Bars = means ± SD (n =4), ^**^, *P* < 0.01. **(B)** The BRCA dataset from the TCGA database was downloaded, and the expression levels of miR-93, CD31 and Emcn in each sample was recorded and analyzed for correlations.

Aiming at the role(s) of miR-93-5p in TNBC angiogenesis, we conducted the CD31 staining of the TNBC tissues, and found that the samples with high miR-93-5p expression had more vessels (Figure 1A). Furthermore, we analyzed The Cancer Genome Atlas (TCGA) database for breast invasive carcinoma (BRCA) to find the correlation of miR-93 expression with the expression of blood vessel markers. As shown in Figure 1B, miR-93 expression level had positive correlation with the CD31 and Endomucin (EMCN) expression, which were considered to be strictly expressed in endothelial cells. These results indicated that miR-93-5p might play a vital role in angiogenesis.

### miRNA-93-5p enhanced ECs proliferation and migration *in vitro*

**Figure 2:**
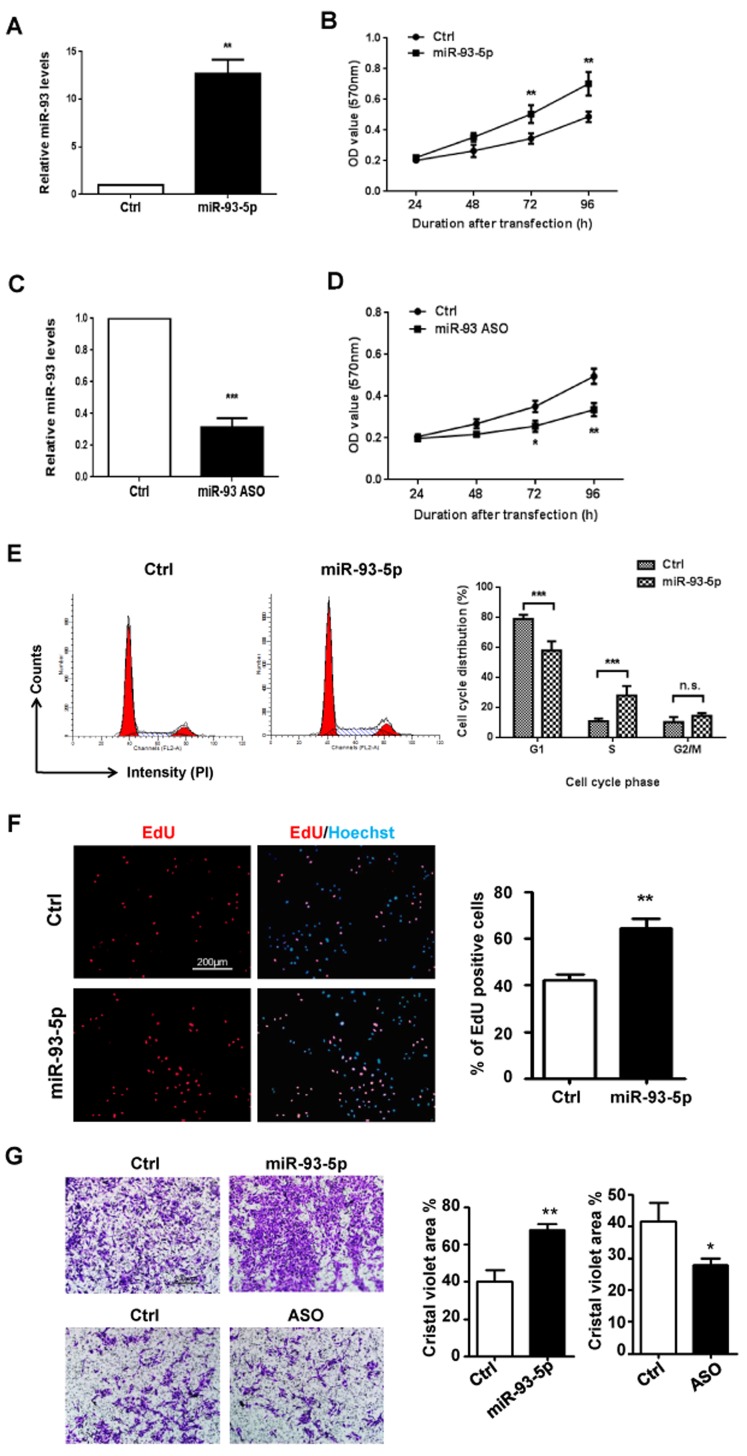
miR-93-5p enhanced ECs proliferation and migration *in vitro* **(A) and (B)** HUVECs were transfected with 100 nM miR-93-5p mimics or control oligos. (A) Up-regulation of miR-93-5p was confirmed using qRT-PCR. (B) Cell viability was determined by the MTT assay. **(C and D)** HUVECs were transfected with 100 nM miR-93-5p ASO or control oligos. (C) qRT-PCR was performed to validate knock-down of miR-93-5p. (D) Cell viability was determined by the MTT assay. **(E)** HUVECs were transfected with 100 nM miR-93-5p or control oligos. Cell cycle distribution was analyzed by using PI staining flow cytometry. Left panel: representative histograms. Right panel: quantification of percentage of cells at each phase of a cell cycle. **(F)** HUVECs were transfected with 100 nM miR-93-5p mimics or ASO and corresponding control oligos. An EdU incorporation assay was performed using a fluorescence microscope. The percentage of proliferating cells was defined as the ratio of EdU positive cells to total cells determined by the Hoechst staining. Left panel: representative florescent images. Right panel: quantification of the percentage of EdU positive cells. **(G)** HUVECs were transfected as described in (F). The capacity of cell migration was measured using a transwell assay. Cells that transmigrate to the lower side of the chamber were stained and quantified. Top panel: representative figures. Bottom panel: quantification of areas stained by crystal violet over total areas. All bars = means ± SD of at least three independent experiments, ^*^, *P* < 0.05, ^**^, *P* < 0.01.

We then employed *in vitro* assays to examine the effect(s) of miR-93 overexpression on ECs. HUVECs were transfected with miR-93-5p mimics or control oligos and the overexpression of miR-93-5p was validated by qRT-PCR (Figure 2A). As shown in Figure 2B, up-regulated miR-93-5p levels significantly accelerated the growth of HUVECs. To deplete miR-93-5p, the anti-sense oligo (ASO) of miR-93-5p or control oligo were transfected into HUVECs and inhibition of miR-93-5p was confirmed by qRT-PCR (Figure 2C). The MTT assay revealed that inhibition of miR-93-5p led to decreased cell viability in HUVECs (Figure 2D). To reveal the mechanisms contributing to the increased cell viability induced by up-regulation of miR-93-5p, we examined the alterations in cell cycle. The results indicated that overexpression of miR-93-5p enhanced the entrance of HUVECs into the S/G2/M phases of cell cycle, suggesting miR-93-5p could accelerate the cell cycle progression (Figure 2E). Furthermore, the results of EdU (5-ethynyl-2’-deoxyuridine) incorporation assay indicated that transfection of miR93-5p mimics increased the number of EdU-positive HUVECs compared to cells transfected with control oligos (Figure 2D), suggesting that miR-93-5p also promoted HUVECs proliferations. Endothelial cell migration is essential to angiogenesis. We therefore detected the effect of miR-93-5p on the migration capacity of ECs. The transwell assay indicated that, compared to cells trasfected with control oligos, a larger number of HUVECs trasfected with miR-93-5p mimics migrated to the lower side of the membrane (Figure 2G), suggesting that miR-93-5p enhanced the migration capacity of HUVECs. In together, these results suggest that miR-93-5p could promote ECs proliferation and migration *in vitro*.

### miRNA-93-5p promoted ECs lumen formation and angiogenic sprouting *in vitro*

**Figure 3:**
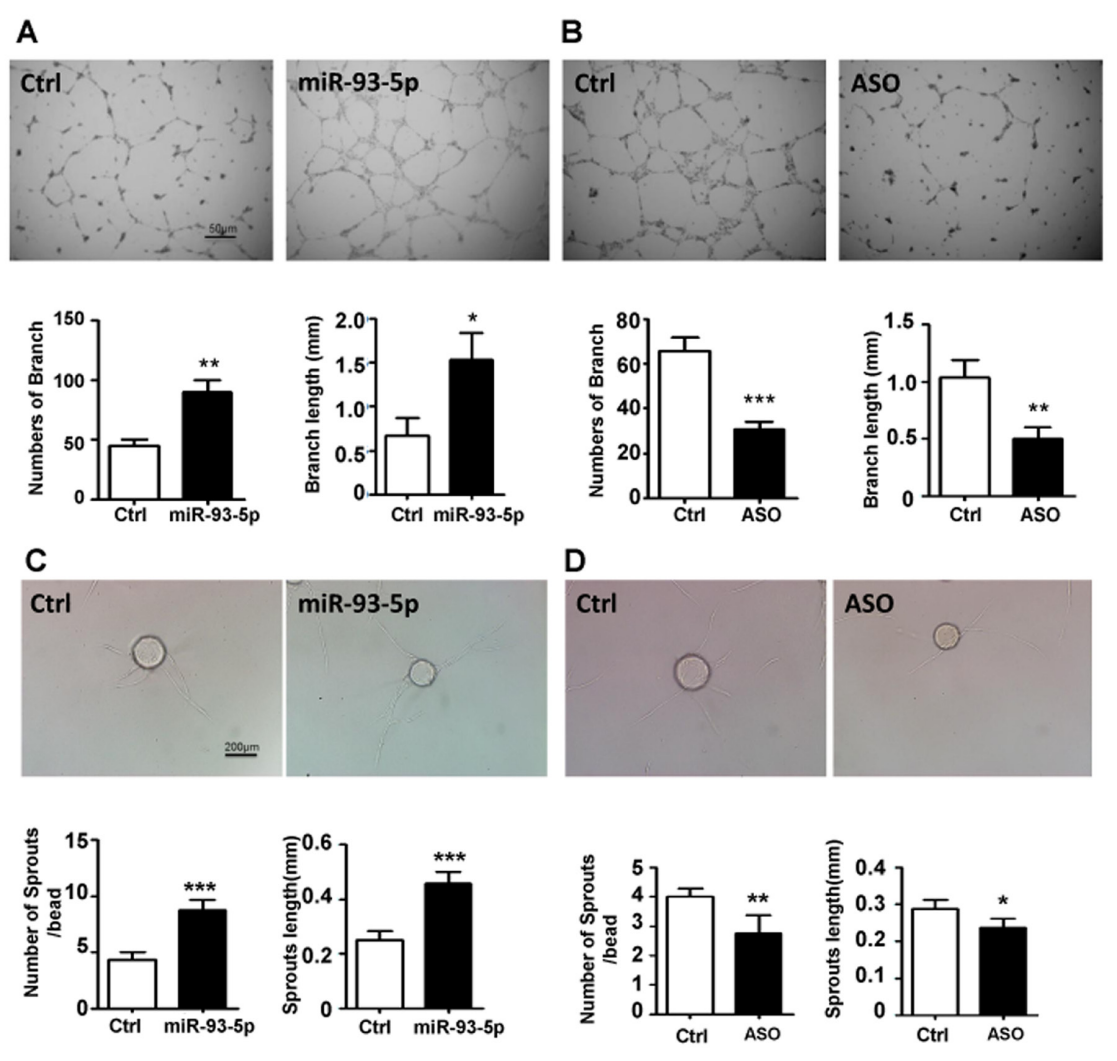
miR-93-5p promoted ECs lumen formation and sprouting *in vitro* **(A and B)** HUVECs were transfected with miR-93-5p mimics (A), miR-93-5p ASO (B), or corresponding control oligos; 48h after transfection the cells were digested and seeded on the matrigel. The number and length of branches were analyzed and compared between the two groups. Top panel: representative figures. Bottom panel: quantified number and length of branches. **(C and D)** HUVECs were transfected as described above. Cells were trypsinized 24h after the transfection and tested for sprouting capacity with a fibrin gel assay. Images were captured and the number and length of sprouts were calculated and compared between the two groups. Top panel: representative figures. Bottom panel: quantified number and length of sprouts. All bars = means ± SD of 4 independent experiments, ^*^, *P* < 0.05, ^**^, *P* < 0.01, ^***^, *P* < 0.001.

HUVECs were transfected with miR-93-5p mimics, ASO and corresponding negative controls, respectively, and subjected to the lumen formation assay. The results showed that miR-93-5p overexpression increased the number and the length of branches (Figure 3A), while blockade of miR-93-5p inhibited the HUVECs lumen formation in terms of both number and length on the matrigel matrix (Figure 3B). We further detected the effects of miR-93-5p on angiogenic sprouting capacity of HUVECs by performing a fibrin gel beads sprouting assay. HUVECs transfected with miR-93-5p mimics formed a larger number of sprouts with longer length compared to the control cells (Figure 3C). However, miR-93-5p inhibitory oligos suppressed the angiogenic sprouting by decreasing both the number and the length of sprouts (Figure 3D). In together, these results indicated that miR-93-5p could promote angiogenesis *in vitro*.

### EPLIN was a direct target of miR-93-5p

**Figure 4:**
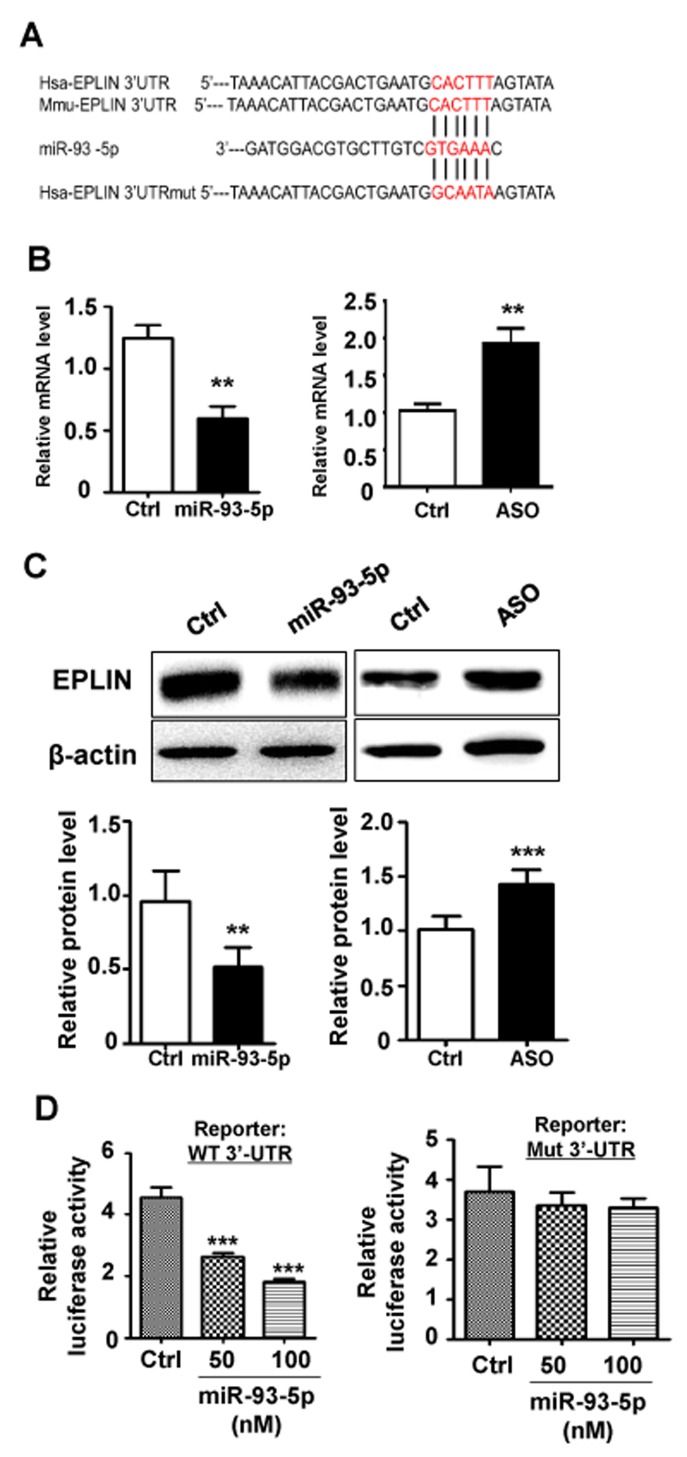
EPLIN was identified as a direct target of miR-93-5p **(A)** the 3’ UTR of human and mouse EPLIN mRNA were aligned with miR-93-5p. The complementary sequence was marked in red. The 3’ UTR of human EPLIN mRNA with mutated miR-93-5p targeting sequence was also shown. **(B)** HUVECs were transfected with miR-93-5p mimics (left panel), miR-93-5p ASO (right panel), or corresponding controls. The changes in the mRNA level of EPLIN were detected by qRT-PCR. **(C)** HUVECs were transfected as described in (B), and the protein levels of EPLIN were determined by a western-blot assay. Bands were quantitatively compared between groups. **(D)** HeLa cells were cotransfected with 100 ng of pGL3-EPLIN-WT or pGL3-EPLIN-mt, together with miR-93-5p mimics (50, 100 nM) or control oligos, and pRL-TK plasmid (5 ng). Cells were collected 48 h after transfection, and the luciferase activity was analyzed. All bars = means ± SD of at least 3 independent experiments, ^*^, *P* < 0.05, ^**^, *P* < 0.01, ^***^, *P* < 0.001.

EPLIN is a cytoskeleton-associated protein involved in regulating actin dynamics and cell motility. Sanders *et al.* showed that over-expression of EPLIN in HECV endothelial cells resulted in a significant reduction in cell migration and tubule formation [16]. The sequence of 3’-UTR of *EPLIN* mRNA is recognizable by the seed region of miR-93-5p (Figure 4A). EPLIN has been predicted as a target of miR-93-5p by multiple bioinformatics tools, including TargetScan, microRNA.org and miRDB. In addition, EPLIN has been experimentally validated as a target of miR-93-5p by several CLIP-based high throughput experiments. To establish the regulatory association between miR-93-5p and EPLIN, we initially measured the expression levels of EPLIN in response to changes in miR-93-5p levels. The results of qRT-PCR revealed that transfection of miR-93-5p mimics reduced the mRNA level of *EPLIN*, while inhibition of miR-93-5p by transfection with ASO up-regulated *EPLIN* mRNA levels (Figure 4B). The western blots assay further confirmed this result, showing that transfection of miR-93-5p mimics and ASO led to reduction and increase in EPLIN protein levels, respectively (Figure 4C). To investigate whether EPLIN was a direct target of miR-93-5p, we constructed luciferase reporters containing the 3’-UTR sequence of *EPLIN* mRNA with either the wildtype or the mutant seed region of miR-93-5p (Figure 4A). The reporter assay further confirmed that miR-93-5p repressed EPLIN expression by directly combining with the 3’-UTR of *EPLIN* mRNA (Figure 4E), and this inhibitory effect was eliminated when the recognition site was disrupted by mutations (Figure 4E).

### miR-93-5p promoted cell migration and angiogenesis through targeting EPLIN

**Figure 5:**
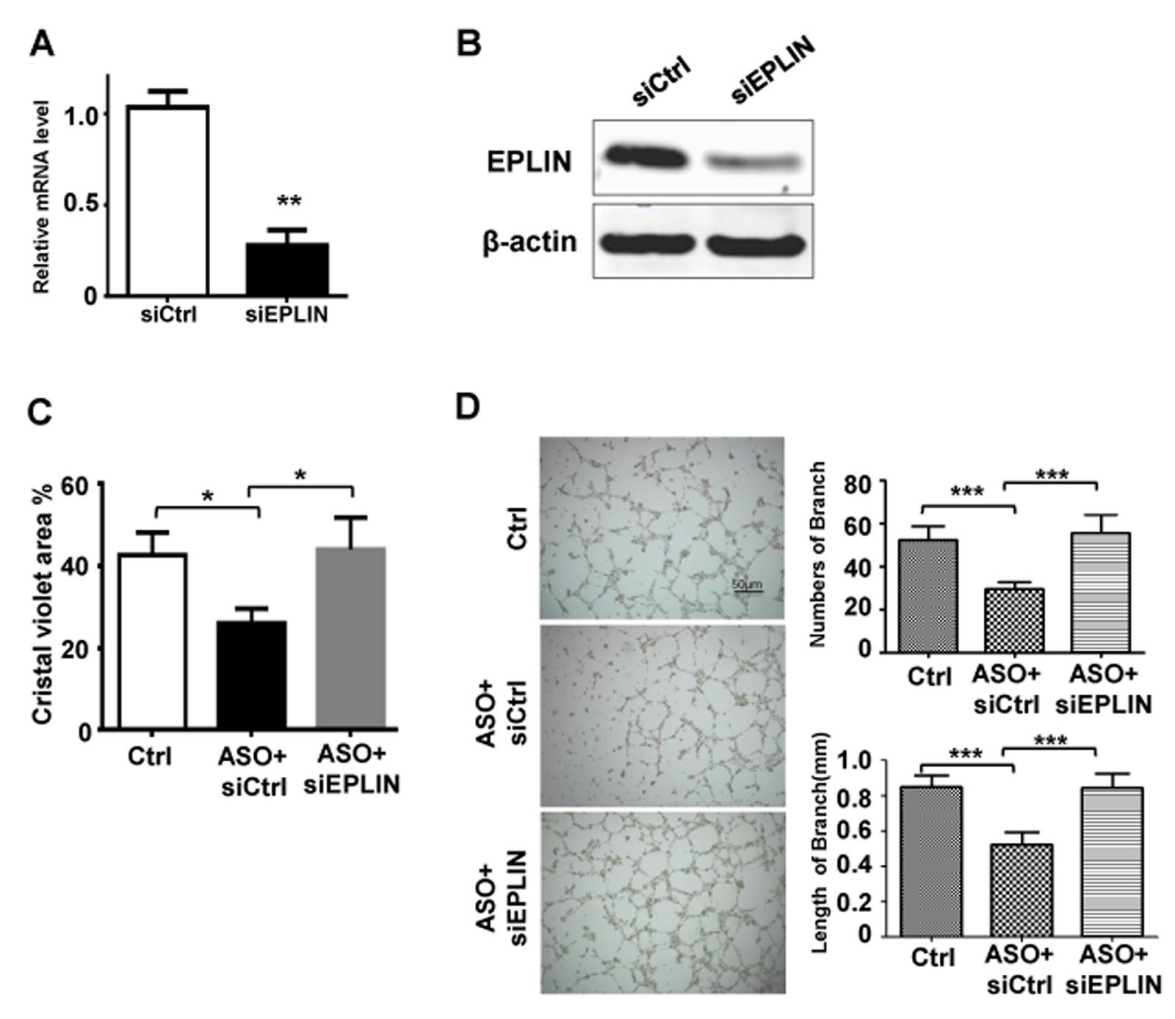
miR-93-5p Promoted Cell Migration and Angiogenesis through Targeting EPLIN **(A and B)** HUVECs were transfected with 50 nM EPLIN specific siRNA or non-targeting siRNA for 48h. (A) The mRNA levels of EPLIN were measured using qRT-PCR. (B) Protein levels of EPLIN were determined using an immunoblotting assay. **(C and D)** HUVECs were transfected with control oligos, miR-93-5p ASO plus non-targeting siRNA, or miR-93-5p ASO mixed with siEPLIN. (C) The cell migration capacity was analyzed using a transwell assay. (D) Cells were seeded on the matrigel for lumen formation assay. Images were captured and the number and length of branches were quantitatively compared among groups. All bars = means ± SD of 3 independent experiments, ^*^, *P* < 0.05, ^**^, *P* < 0.01, ^***^, *P* < 0.001.

We further investigated whether the effects of miR-93-5p on HUVECs migration and angiogenesis was through down-regulating EPLIN. A siRNA specifically targeting EPLIN led to decreased mRNA and protein levels of EPLIN in HUVECs (Figure 5A and 5B). We also found that depletion of EPLIN by transfection of specific siRNA could attenuate the effects of miR-93-5p ASO on the ECs migration (Figure 5C) and lumen formation (Figure 5D), further confirming that miR-93-5p could regulate angiogenesis by directly targeting the *EPLIN transcripts*.

## DISCUSSION

Tumor angiogenesis is essential for almost all steps in cancer formation, aggression, and metastasis and thus has been considered as a hallmark of cancer [1]. Anti-angiogenesis has become an effective anti-cancer strategy in several types of cancers, such as colorectal cancer, kidney cancer and liver cancer [2, 4–6]. Unfortunately, the anti-angiogenic reagents are effective only in specific cancer types and drug resistance develops over time. Therefore, a better understanding of the molecular basis of tumor angiogenesis is critical for developing novel therapeutic strategies and reversing drug resistance.

Recent publications demonstrated that miR-93-5p is frequently up-regulated in multiple types of cancers, including breast cancer [10], osteosarcoma [11] and gastric cancer [12] and functions as an onco-miRNA in tumor growth [10–12]. Accumulating evidence suggests that miRNAs participate in modulating angiogenesis through regulating the expression levels of a variety of angiogenesis-related genes [9]. However, the role(s) of miR-93-5p in regulating angiogenesis remain largely underexplored. In this study, we demonstrate that the TNBC samples with high miR-93-5p expression had higher levels of blood vessels marker CD31 staining (Figure 1A), suggesting miR-93-5p might be involved in angiogenesis regulation. This result was consistent with the findings in gliomas [13–15]. Fang *et al.* reported that miR-93-5p promoted tumor angiogenesis and metastasis by suppressing LATS2 expression in astrocytoma [13]. In another study, Fang *et al.* showed that miR-93 promoted astrocytoma growth and angiogenesis by down-regulating integrin-β8 [14]. Fabbri *et al.* reported that miR-93 regulated the secretion of a panel of cytokines, chemokines and growth factors that were involved in angiogenesis in gliomas through suppressing IL-8 and VEGF [15]. However, these assays were performed based on the co-culture of tumor cells ectopically over-expressing miR-93-5p with endothelial cells. No literature has reported the role(s) of miR-93-5p in regulating the angiogenic capacity of endothelial cells directly. Therefore, we investigated the direct role(s) of miR-93-5p in regulating the angiogenesis capacity of HUVECs.

Initially, we investigated the effects of miR-93-5p on HUVECs growth *in vitro*. We found that up- and down-regulated miR-93-5p levels significantly accelerated and decreased the growth of HUVECs, respectively (Figure 2B and 2D). Further studies revealed that over-expression of miR-93-5p enhanced cell cycle progression into the S/G2/M phases (Figure 2E) and promoted HUVECs proliferations (Figure 2F). The transwell assay indicated that miR-93-5p enhanced the migration capacity of HUVECs, which is essential to angiogenesis (Figure 2G). These functions were in consistent with the oncogenic functions of miR-93-5p in cancer cells, including promoting tumor growth and cell cycle progression. We then investigated the effects of miR-93-5p on blood vessel generation by measuring HUVECs lumen formation and angiogenic sprouting *in vitro*. The results revealed that miR-93-5p overexpression increased the number and length of branches (Figure 3A), while suppression of miR-93-5p inhibited the HUVECs lumen formation in terms of both number and length on the matrigel matrix (Figure 3B). Furthermore, the fibrin gel beads sprouting assay revealed that miR-93-5p promoted the angiogenic sprouting capacity of HUVECs by increasing the number and length of sprouts (Figure 3C). In together, these results indicated that miR-93-5p could promote angiogenesis *in vitro*. These results were in consistence with the co-culture based studies in gliomas as mentioned above. However, to further confirm the results of *in vitro* studies, animal-based *in vivo* experiments are guaranteed in further studies.

MiRNAs exert functions through regulating target mRNA transcripts. As a candidate target of miR-93-5p, ELPIN was down-regulated by miR-93-5p which bonded to its target site within the 3’UTR of ELPIN (Figure 4). EPLIN is a cytoskeleton-associated protein encoded by the *LIMA1* gene, involved in regulating actin dynamics by cross-linking and stabilizing filaments and is involved in regulation of cell motility [18]. EPLIN expression is often decreased in a variety of cancers, including breast cancer, prostate cancer and esophageal cancer [18]. The loss of EPLIN may contribute to the increased invasive phenotype in cancer cells. Not surprisingly, EPLIN also appeared to show an anti-angiogenic role *in vitro*. Sanders *et al.* showed that over-expression of EPLIN in HECV endothelial cells resulted in a significant reduction in cell migration and tubule formation [16]. Interestingly, ELPIN was also proved to stabilize vascular capillary network *in vitro* by interacts with α-catenin and actin filaments in endothelial cells. With this characteristic, endothelial cells depletion of ELPIN exhibited a reduced capacity to form pseudocapillary networks and more leakage. We found that depletion of EPLIN by transfection of specific siRNA could attenuate the inhibitory effect of miR-93-5p ASO on the HUVECs migration (Figure 5C) and lumen formation capacities (Figure 5D), further confirming that miR-93-5p could promote angiogenesis by directly targeting EPLIN. These results suggested that miR-93-5p might enhance angiogenesis and reduce vascular capillary stabilization by targeting ELPIN and that both miR-93-5p and EPLIN represented promising targets for developing novel anti-angiogenic therapeutics. Notably, a specific miRNA usually targets a variety of mRNA transcripts; therefore, in addition to EPLIN, other target genes of miRN-93-5p also have been reported, such as p21, E2F-1, PTEN, CCNB1 and LATS2 [13, 19–21]. Moreover, some of the miRN-93-5p targets, such as LATS2, are associated with the angiogenic process. Thus, investigations in the role(s) of other potential targets of miRN-93-5p in the angiogenic capacity of ECs are guaranteed in our future studies.

In sum, our findings first demonstrated that miR-93-5p enhanced angiogenesis and reduced vascular capillary stabilization by targeting ELPIN in HUVECs. Further studies are guaranteed to fully understand the roles of miR-93-5p in regulating tumor angiogenesis, which include the expression levels of miR-93-59 in endothelial cells of tumor blood vessels and animal model-based *in vivo* studies. We believe that this study contributed to the understanding of miR-93-5p’s biological functions, shed new light on the regulation of angiogenesis and provided a potential novel target for developing anti-angiogenic reagents.

## MATERIALS AND METHODS

### Human tissues

Human TNBC tissues and human umbilical cord biopsies were collected from the Department of Oncology and the Department of Obstetrics and Gynecology, respectively, of the Second Affiliated Hospital Xi’an Jiaotong University. All human participants in the study had signed informed consent for the use of their tissue samples according to the protocols approved by the Ethics Review Board at the Medical School of Xi’an Jiaotong University.

### Analysis of the BRCA dataset in TCGA database

The BRCA dataset for 1097 invasive breast cancer samples was obtained from TCGA. Level 3 miRNA-Seq data for miR-93 and mRNA-seq data for CD31 and EMCN were retrieved from the TCGA Data Portal. The correlations between the expression of miR-93 and CD31 or EMCN were calculated using the Pearson’s correlation analysis. As the data were obtained from TCGA, further approval by an ethics committee was not required. This study meets the publication guidelines provided by TCGA.

### Cell culture and transfection

Primary HUVECs were isolated and characterized by staining with CD31 and photographing under a fluorescence microscope as described previously [22]. The purity of HUVECs was close to 95% (Supplementary Figure 1). HUVECs were maintained in the EC medium (ScienCell, USA) supplemented with 5% FBS and EC growth supplements at 37°C in a humidified atmosphere containing 5% CO_2_. Cells at passages between 2 and 5 were used in all experiments. HUVECs were transfected with 100 nM of miR-93-5p mimic oligos (RiboBio, China) or miR-93-5p ASO (RiboBio, China) by using Lipofectamin 2000 reagent (Invitrogen, USA) according to manufacture’s instructions. HUVECs transfected with respective non-targeting oligos were used as controls. HeLa cells were obtained from Peking Union Medical Center Laboratory (Beijing, China) and cultured in Dulbecco’s modified Eagle’s medium (DMEM) supplemented with 10% fetal calf serum (FCS) at 37°C in a humidified atmosphere containing 5% CO_2_.

### The MTT assay

The MTT assay was performed to measure cell viability as described before [23]. After transfection, HUVECs were inoculated in 96-well plates at 2500 cells/well in quadruplicate and incubated at 37°C. At different time points (24, 48, 72 or 96 h), 10 μl MTT solution (5 mg/mL; Sigma, USA) was added to each well, and the plates were incubated at 37°C for another 4 h. Then, 100 μl SDS-HCl (10%) solubilization buffer was added to each well and incubated for overnight. The absorbance at 570 nm wavelength was measured by using a microplate reader. Three independent experiments were performed for each treatment.

### Cell cycle analysis

HUVECs were trypsinized, washed with ice-cold PBS, and fixed in 75% ethanol at 4°C for at least 2 hrs. The collected cells were treated in PBS containing 0.02% Triton X-100, 3000 U/mL RNase A and 50 μg/mL propidium iodide for 30 minutes at 37°C. Subsequently, the cell cycle was investigated with flow cytometry by using a FACSCalibur flow cytometer (BD Immunocytometry Systems, USA). Data analyses were performed using the ModFit LT software (Verity Software House, USA).

### Cell proliferation assay

Cell proliferation was detected by using the Cell-Light EdU proliferation assay kit (RiboBio, China) according to manufacture’s instructions. Briefly, HUVEC cells were cultured in EC medium containing 50 μM Apollo 567-conjugated EdU (5-ethynyl-20- deoxyuridine) for 2 hrs and then fixed with 4% paraformaldehyde for 20 min. The fluorescent signal was detected and captured by an Olympus BX51 fluorescence microscopy (Olympus, Japan). More than 5 random fields per well were captured at a magnification of 100 and the percentage of EdU-positive cells was calculated as Apollo 567 fluorescence positive cells in total cells identified by Hoechst 33342 nuclear staining.

### Cell migration assay

Confluent HUVECs monolayers were trypsinized and seed in the Transwell chamber (Millipore, USA) placed in 24-well plates and cells were cultured to migrate to the lower side of the polycarbonate membrane in complete medium for 24 hrs. Cells on the lower side were fixed with 4% paraformaldehyde and stained with crystal violet and the number of stained cells was counted under a microscope.

### Endothelial lumen formation assay

For the lumen formation assay, 48 well plates were pre-coated with 200 μl of the Matrigel Basement Membrane Matrix (BD Biosciences, USA). HUVECs were then seeded on the wells and cultured at 37°C. After 8 hours, images were photographed under a microscope and the number of branches and the length of the cell cords of the enclosed lumens were determined.

### Fibrin gel beads sprouting assay

Fibrin beads sprouting assay was conducted according to the standard protocol. Briefly, HUVECs were digested and incubated with the Cytodex 3 microcarrier beads (Sigma, USA) at a density of 400 cells per bead at 37 °C for overnight. The microbeads were then mixed with fibrinogen (Sigma, USA) containing 0.625 U/ml thrombin (Sigma, USA) at a density of 100 beads/ml and embedded in a 48-well plate, and 0.5 ml of EGM-2 medium (Clonetics, USA) containing with lung fibroblasts (20,000 cells/well) was added. The medium was changed every other day for 4 days. Images of the beads were captured under a microscope (CKX41, Olympus, Japan), and the number and length of sprouts were measured.

### Western blot analysis

Western blots was performed as previously described [24]. Cells were lysed in the RIPA buffer (Beyotime, China), and the protein concentration was measured by using a BCA Protein Assay kit (Pierce, USA). Totally 30 μg cell lysates were separated by sodium dodecyl sulfate-polyacrylamide gelelectrophoresis (SDS-PAGE), blotted onto polyvinylidene fluoride (PVDF) membranes, which were blocked with 5% non-fat dried milk in TBS-T and then probed with primary antibodies. Then the membranes were incubated with horseradish peroxidase (HRP)-conjugated goat anti-rabbit IgG or goat anti-mouse IgG (Boster Bio Tec, China). β-actin was used as a loading control. The primary antibodies included rabbit anti-human EPLIN (1:250, Abcam, USA), mouse anti-β-actin (1:1000, Sigma, USA). Membranes were then detected by using the enhanced chemoluminescence (ECL) system (Clinx Science Instruments, China).

### qRT-PCR for EPLIN mRNA levels

The qRT-PCR was performed as previously described [25]. Total RNA was extracted with the TRIzol reagent (Invitrogen, USA) according to the manufacturer’s protocol. Briefly, 1.0 μg total RNA was reverse transcribed into cDNA using a PrimeScript RT Reagent Kit (Takara Bio, China) in a 20 μl reaction system according to the manufacturer’s protocol. Real-time PCR was conducted by using a SYBR Premix Ex Taq Kit (Takara Bio, China) on an ABI PRISM 7500 Real-time PCR system (Life Technologies, USA). qRT-PCR cycling conditions were 95 °C for 30 sec, followed by 40 cycles of 95 °C for 5 sec and 60 °C for 30 sec. Glyceraldehyde-3-phosphate dehydrogenase (GAPDH) as used as an internal control. The fold-change in EPLIN levels was calculated using the 2-^ΔΔ^CT method. The PCR primers are listed in Supplementary Table 1.

### qRT-PCR for miR-93 expression

Total RNA, including microRNAs, was extracted using a miRNeasy mini kit (Qiagen, USA) according to manufacture’s instructions. The total RNA was converted to cDNA and added with a universal tag by using a miScript II RT kit (Qiagen, USA). To measure the levels of miR-93, a qRT-PCR assay was conducted via using the miScript SYBR Green PCR kit (Qiagen, USA) according to manufacture’s instructions. The following primers were used: miR-93, 5’-CAAAGTGCTGTT CGTGCAGGTAG -3’ and U6 5’-GGATGACACGCAAATTCGTGAAGC -3’. The PCR protocol included 95 °C for 15 min, followed by 40 cycles of 94 °C for 15 sec, 55 °C for 30 sec, and 70 °C for 30 sec. The qRT-PCR was performed in triplicate. The expression level of miR-93 was normalized to U6 snRNA. The fold-change in miR-93 levels was calculated using the 2-^ΔΔ^CT method.

### Luciferase reporter assay

The 3’-UTR of the human EPLIN mRNA (NM_001114676.1) was amplified from cDNA derived from total RNA of HUVECs, and subcloned into pGL3-promoter vector (Promega, USA) to construct a pGL3-EPLIN-WT reporter plasmid. A mutant reporter (pGL3-EPLIN-mt) with a mutation in the 3’-UTR complementary to the seed sequence of miR-93-5p was generated by using a Site Directed Mutagenesis Kit (Clontech, USA) according to manufacture’s instructions. The PCR primers are listed in Supplementary Table 1. To perform the luciferase reporter assay, HeLa cells were cotransfected with 100 ng of pGL3-EPLIN-WT or pGL3-EPLIN-mt, together with miR-93-5p mimics (50, 100 nM) or control oligos, and pRL-TK (5 ng). Cells were collected 48 h after the transfection, and firefly and renilla luciferase activities were analyzed with the Dual-Luiferase Reporter Assay System (Promega, USA) according to the manufacturer’s instruction.

### Immunofluorescence

HUVECs were grown on a cover slide until confluent and then fixed with 4% paraformaldehyde. Tumor cryosections or the fixed HUVECS were blocked and permeabilized in PBS containing 1% bovine serum albumin (BSA) and 0.3% TritonX-100, then immunostained with rat anti-CD31 (1:400, BD, USA) antibody at 4°C for overnight. After washing with PBS, sections were incubated with FITC-conjugated goat anti-rat secondary antibody(1:500, Abcam, USA) at room temperature for 2 hours, followed by Hoechst staining. Images were captured with a fluorescence microscope.

### Statistics

The Image Pro Plus 6.0 was used to quantify images. All data analysis was performed using Graph Pad Prism5 software (GraphPad Software, USA). Student’s *t* test was used to compare means of two groups. The *one-way* ANOVA followed by Tukey *post hoc* analysis was carried out to compare means of more than two groups. Data were presented as the means ± SD of at least three independent experiments. A two-tailed P < 0.05 was considered to be statistically significant.

## SUPPLEMENTARY MATERIALS FIGURE AND TABLE



## Abbreviations

3’UTR: 3’ untranslated region
TNBC: triple negative breast cancer
HUVECs: human umbilical vein endothelial cells
EPLIN: epithelial protein lost in neoplasm
TCGA: The Cancer Genome Atlas
