# Supplementary material for: MiR-93-5p enhances growth and angiogenesis capacity of HUVECs by down-regulating EPLIN
Source: Oncotarget. 2017 Nov 6;8(63):107033–43. doi: 10.18632/oncotarget.22300 (PMC5739794; doi:10.18632/oncotarget.22300)
Supplement: Supplementary file 1 [file oncotarget-08-107033-s001.pdf]

MiR-93-5p enhances growth and angiogenesis capacity of HUVECs by down-regulating EPLIN

SUPPLEMENTARY MATERIALS

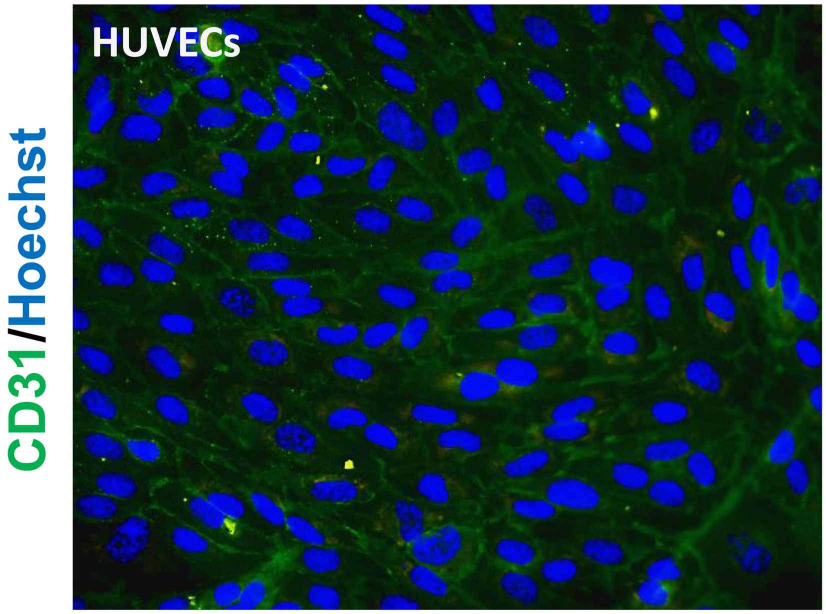

Supplementary Figure 1: The immunostaining for CD31 expression in cultured HUVECs. HUVECs were grown on a cover slide until confluent and then immunostained for CD31, followed by Hoechst staining. Images were captured with a fluorescence microscope. The image was a representative image of CD31 staining.

Supplementary Table 1: Sequences of primers and oligonucleotides

Primer Name	Sequence
miR-93-5p (qPCR)	5'-CAAAGTGCTGTTCGTGCAGGTAG -3'
U6 (qPCR)	5'-GGATGACACGCAAATTCGTGAAGC -3'
Human *eplin* forward (qPCR)	5'-GACTCCCAGGTTAAGAGTGAGG -3'
Human *eplin* reverse (qPCR)	5'-TTGCAGGTGCCTGAAACTTCT -3'
Human *eplin* 3'-UTR forward (Clone)	5'-GAATTCTGCAATGATGCTGGGCCTTA -3'
Human *eplin* 3'-UTR reverse (Clone)	5'-CTGCAGAGACACCTCTCCCTCCCATC -3'
Human *eplin* 3'-UTR mt forward (Clone)	5'-CGTTATTCATATTTCCCGTGTC -3'
Human *eplin* 3'-UTR mt reverse (Clone)	5'-TATTGCCATTCAGTCGTAATG -3'
